# Stellettin B-Induced Oral Cancer Cell Death via Endoplasmic Reticulum Stress–Mitochondrial Apoptotic and Autophagic Signaling Pathway

**DOI:** 10.3390/ijms23158813

**Published:** 2022-08-08

**Authors:** Tsu-Jen Kuo, Yen-Hsuan Jean, Po-Chang Shih, Shu-Yu Cheng, Hsiao-Mei Kuo, Yi-Ting Lee, Yu-Cheng Lai, Chung-Chih Tseng, Wu-Fu Chen, Zhi-Hong Wen

**Affiliations:** 1 Department of Marine Biotechnology and Resources, National Sun Yat-sen University, Kaohsiung 80424, Taiwan; 2 School of Dentistry, Chung Shan Medical University, Taichung 40201, Taiwan; 3 Department of Dentistry, Chung Shan Medical University Hospital, Taichung 40201, Taiwan; 4 Section of Orthopedics, Department of Surgery, Antai Medical Care Corporation Antai Tian-Sheng Memorial Hospital, Pingtung 92842, Taiwan; 5 Department of Neurosurgery, Kaohsiung Chang Gung Memorial Hospital and Chang Gung University College of Medicine, Kaohsiung 83301, Taiwan; 6 Department of Environmental Protection, Green Technology Research Institute, CPC Corporation, No. 2, Zuonan Rd., Nan-Tzu District, Kaohsiung 81126, Taiwan; 7 Department of Orthopedics, Asia University Hospital, Taichung 41354, Taiwan; 8 Zuoying Branch of Kaohsiung Armed Forces General Hospital, Kaohsiung 80284, Taiwan; 9 Institute of Medical Science and Technology, National Sun Yat-sen University, Kaohsiung 80424, Taiwan

**Keywords:** stellettin B, autophagy, mitochondrial stress, ER stress, BiP/GRP78

## Abstract

Oral squamous cell carcinoma (OSCC) affects tens of thousands of people worldwide. Despite advances in cancer treatment, the 5-year survival rate of patients with late-stage OSCC is low at 50–60%. Therefore, the development of anti-OSCC therapy is necessary. We evaluated the effects of marine-derived triterpene stellettin B in human OC2 and SCC4 cells. Stellettin B dose-dependently decreased the viability of both cell lines, with a significant reduction in OC2 cells at ≥0.1 µM at 24 and 48 h, and in SCC4 cells at ≥1 µM at 24 and 48 h. Terminal deoxynucleotidyl transferase dUTP nick-end labeling (TUNEL)-positive cells were significantly observed at 20 µM of stellettin B at 48 h, with the overexpression of cleaved caspase3 and cleaved poly(ADP-ribose) polymerase (PARP). Moreover, mitochondrial respiratory functions were ablated by stellettin B. Autophagy-related LC3-II/LC3-I ratio and Beclin-1 proteins were increased, whereas p62 was decreased. At 20 µM at 48 h, the expression levels of the endoplasmic reticulum (ER) stress biomarkers calnexin and BiP/GRP78 were significantly increased and mitogen-activated protein kinase (MAPK) signaling pathways were activated. Further investigation using the autophagy inhibitor 3-methyladenine (3-MA) demonstrated that it alleviated stellettin B-induced cell death and autophagy. Overall, our findings show that stellettin B induces the ER stress, mitochondrial stress, apoptosis, and autophagy, causing cell death of OSCC cells.

## 1. Introduction

Oral squamous cell carcinoma (OSCC) is a common cancer across the globe, with ~650,000 new cases and 330,000 deaths annually according to the *World Cancer Report*. In Asia, OSCC remains the third-most common malignancy [1]. OSCC refers to any types of malignant tumors developed in the oral cavity, with oral squamous cell carcinoma accounting for 90% of cases. Surgical resection is the main treatment for patients diagnosed with early-stage OSCC, whereas patients with late-stage OSCC undergo chemotherapy with radiotherapy [2,3]. To date, advances in medical treatments for OSCC have significantly increased the survival rate of patients with early-stage OSCC; however, the 5-year survival rate of patients with late-stage OSCC remains as low as 50–60% [3]. This has prompted the need for developing novel chemotherapeutics for improving the survival rate.

Mitochondria are power plants of a cell; the abnormality of these organelles leads to multiply defects such as loss of redox control, cell homeostasis, and may ultimately cell death. The oxidative phosphorylation (OXPHOS) reaction on the mitochondrial membrane is a tightly regulated energy provision process which enzymatic complexes I-V are involved, and it is free radical rich that could cause leakage of free radicals. An excess of free radicals leakage could induce mitochondrial autophagy (also known as mitophagy), ultimately causing mitophagic cell death [4,5,6]. This type of cell death has thus been researched to develop cytotoxic chemotherapeutics for cancer diseases.

The endoplasmic reticulum (ER) functions as an important sensory organelle, which can coordinate stress-related signaling pathways that are vital in maintaining the crosstalk between the intracellular and extracellular environments of cancer cells. ER stress is caused by physiology and pathology, such as the accumulation of chemotherapeutics and misfolded proteins [7,8]. Nevertheless, under prolonged stress, such as ER stress, autophagy could be overactivated, ultimately resulting in autophagic cell death [9]. Autophagy is a self-degradative and self-recycling process that maintains cellular homeostasis. As a response to cellular stress, self-degradation is critical for preventing nutrient starvation and cytotoxicity-induced injuries and promoting cell survival. Autophagy provides cytoprotection by degrading and clearing unfolded proteins and damaged organelles [10,11]. Although autophagy was initially identified as a cell survival and protection mechanism, it plays a specific role in modulating cell death of a cell. There is an interaction between autophagy-dependent and other types of cell death [12,13]. Recent studies have shown that mitogen-activated protein kinase (MAPK) plays a vital role in cancer progression, involving multiple cellular responses, such as balancing apoptosis, inducing autophagy, and inducing autophagic cell death [14,15].

Stellettin B, a yellow-colored isomalabaricane triterpene, was originally isolated from the sponge *Jaspis stellifera* [16,17]. In our previous study, stellettin B inhibited invasion and angiogenesis in glioblastoma [18]; in addition, we found that stellettin B at as low a concentration as 0.1 nM yields neuroprotection by regulating the nuclear factor erythroid 2-related factor 2/heme-oxygenase 1 (Nrf2/HO-1) pathway in vitro and in vivo [19]. Other studies have also reported that stellettin B shows cytotoxicity and apoptosis against human glioblastoma, non-small-cell lung cancer, and chronic myeloid leukemia [20,21,22,23]. However, the role of stellettin B in regulating mitochondrial stress, ER stress, and autophagy in OSCC cells remains unknown. This study aimed to investigate the mechanism underlying mitochondrial and ER stress resulting from the addition of stellettin B to OSCC cell lines OC2 and SSC4.

## 2. Results

### 2.1. Stellettin B Inhibits the Viability of OSCC Cells

**Figure 1:**
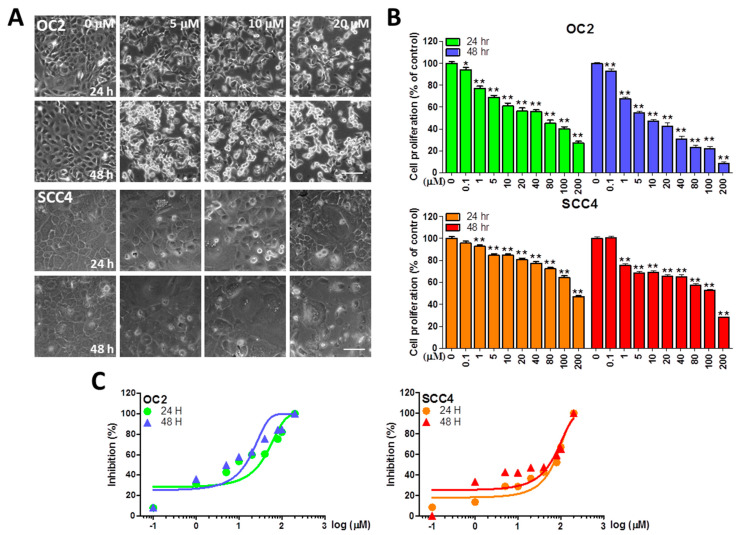
The effects of stellettin B on cytotoxicity in OC2 and SCC4 cells. (**A**) Morphological images of OC2 and SCC4 cells after exposure to 0, 5, 10, or 20 µM stellettin B for 24 and 48 h. Phase-contrast microscopy was performed to observe the cells. Scale bar = 100 μm. (**B**) Stellettin B treatment affected the viability of human oral squamous cell carcinoma (OSCC) cells at 0, 0.1, 1, 5, 10, 20, 40, 80, 100, and 200 µM at 24 and 48 h, using the crystal violet assay. (**C**) The same stellettin B concentrations and inhibition (%) converted in the sigmoidal curve of OC2 and SCC4 cells for 24 and 48 h. Error bars represent the mean ± standard error (SE). The data are shown for a minimum of three independent trials in triplicate analyzed using Student’s *t*-test to determine significance; * *p* < 0.05 and ** *p* < 0.01 relative to the control (untreated cells).

To understand the cell viability of the OSCC cell lines OC2 and SCC4 after stellettin B treatment, a crystal violet assay was performed. OC2 and SCC4 cells were treated with stellettin B at 0, 0.1, 1, 5, 10, 20, 40, 80, 100, and 200 µM for 24 and 48 h. Microscopic observation of OC2 and SCC4 cells showed that stellettin B causes morphological changes and significant cell death (Figure 1A). A significant reduction in cell viability was observed in OC2 cells at ≥0.1 µM at 24 and 48 h, and in SCC4 cells at ≥1 µM at 24 and 48 h (Figure 1B). We also converted the same stellettin B concentrations and inhibition (%) in the sigmoidal curve for 24 and 48 h, with 50% inhibitory concentration (IC_50_) estimated to be <40 and <200 µM, respectively, for SCC4 and OC2 cells (Figure 1C). In both cell lines, cell viability decreased with increasing stellettin B concentration, indicating dose dependency of the natural product.

### 2.2. Stellettin B Induces Cell Cycle Arrest and Apoptosis

**Figure 2:**
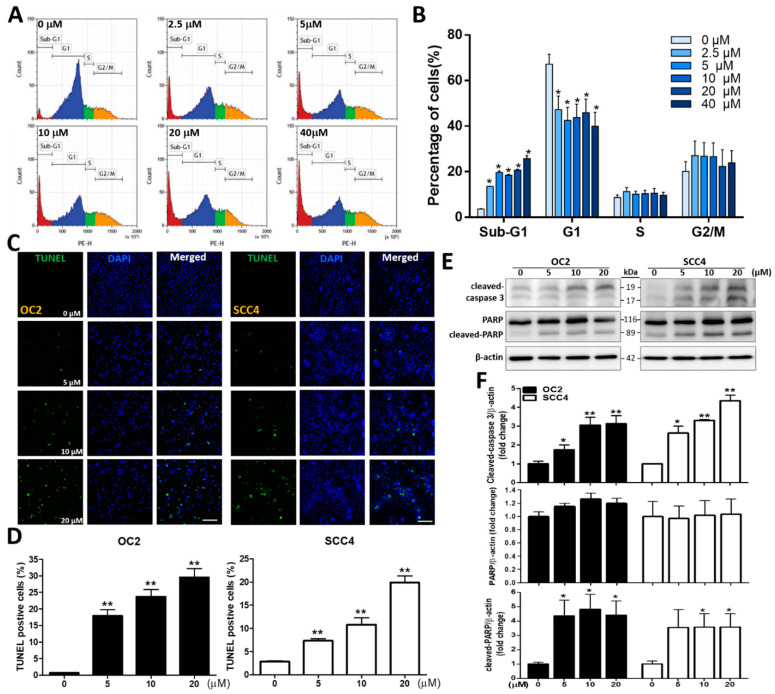
Stellettin B-induced cell cycle arrest and apoptosis in human OSCC cells. (**A**) Analysis of cell cycle distribution of OC2 cells treated with stellettin B was assisted with propidium iodide (PI) staining-based flow cytometry. The cells were exposed to 0, 2.5, 5, 10, 20, and 40 µM stellettin B for 24 h. The peaks in the flowcharts indicate the cell cycle phases of sub-G1, G1, S, and G2/M. (**B**) Histograms displaying the proportion of each phase of the cell cycle under the indicated stellettin B concentration in OC2 cells. (**C**) OC2 and SCC4 cells were exposed to 0, 5, 10, and 20 µM stellettin B for 48 h and then stained with TUNEL dye (emitting green fluorescence) and 4,6-diamidino-2-phenylindole (DAPI) (showing blue fluorescence). The cells were placed under a fluorescence microscope for observation. Scale bar = 100 μm. (**D**) Quantified levels of TUNEL positive cells. (**E**) Western blot analysis of the expression of the apoptosis-related protein, cleaved caspase 3, PARP, and cleaved PARP, in OC2 and SCC4 cells treated with 0, 5, 10, and 20 µM stellettin B for 48 h. (**F**) Quantified levels of cleaved caspase 3, PARP, and cleaved PARP by comparing with β-actin. Original, uncropped images of the Western blots are displayed in Supplementary Figure S1A. β-actin was blotted as an internal control, and error bars stand for the mean ± SE. The data are shown for a minimum of three independent trials in triplicate analyzed using Student’s *t*-test to determine significance; * *p* < 0.05 and ** *p* < 0.01 relative to the control (untreated cells).

A subsequent experiment was conducted to analyze cell cycle distribution, including events of sub-G_1_, G_1_, S, and G_2_/M, of OC2 cells after stellettin B treatment at 0, 2.5, 5, 10, 20, and 40 µM for 24 h. The sub-G1 phase indicates the cell population as a result of apoptosis, while the G1 phase represents preparation for DNA replication. The S phase describes DNA replication, and the G_2_/M phase indicates G_2_-to-M phase transition for cell division [24,25]. Stellettin B treatment at 2.5, 5, 10, 20, and 40 µM significantly induced apoptosis in OC2 cells (Figure 2A,B). To understand whether stellettin B-induced OSCC cell death is associated with apoptosis, we next investigated its effects on it. The terminal deoxynucleotidyl transferase dUTP nick-end labeling (TUNEL)-based method was carried out to detect DNA breakage via labeling 3′-OH ends of double-stranded DNA breaks generated during apoptosis. Our findings showed that 5 µM and above of stellettin B induced significantly TUNEL signal intensity at 48 h compared with the control (Figure 2C,D). Western blot was subsequently used to analyze the cleaved form of poly(ADP-ribose) polymerase (PARP) and caspase 3 (apoptotic biomarkers) which was significantly increased after 48 h of 5 µM and above of stellettin B treatment, compared with the corresponding controls. The fold changes at 5, 10, and 20 µM are 1.6 ± 0.3, 3.1 ± 0.5, 3.1 ± 0.6 for cleaved caspase 3 in OC2 cells, and 2.6 ± 0.4, 3.3 ± 0.1, 4.4 ± 0.3 in SCC4 cells, respectively. The fold changes at 5, 10, and 20 µM are 4.4 ± 2.5, 4.8 ± 2.3, 4.4 ± 2.2 for cleaved PARP in OC2 cells, and 3.5 ± 2.8, 3.6 ± 2.1, 3.6 ± 2.1 in SCC4 cells, respectively (Figure 2E,F). Collectively, these findings indicate that stellettin B treatment significantly enhanced caspase-associated apoptosis in OC2 and SCC4 Cells.

### 2.3. Stellettin B Down-Regulates Oxygen Consumption of Mitochondria

**Figure 3:**
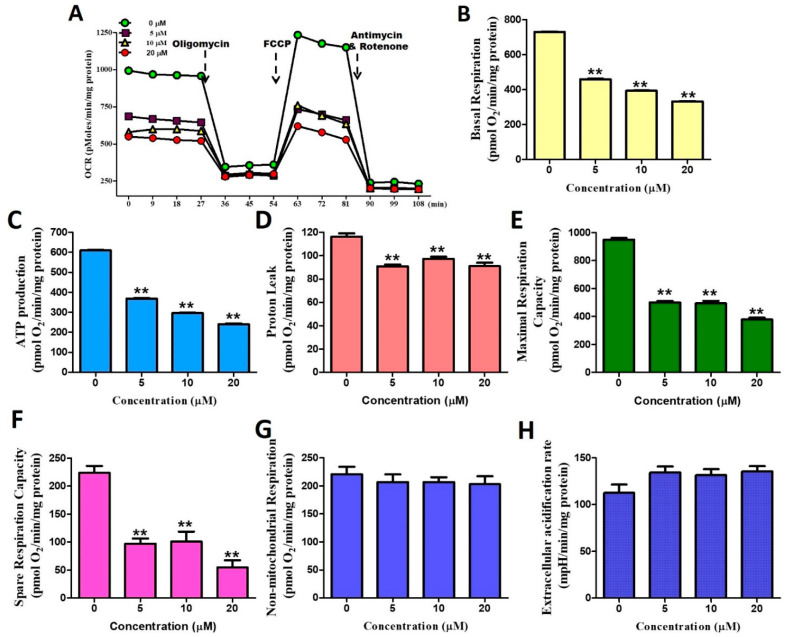
Stellettin B-induced down-regulation of mitochondrial respiration in OC2 cells at 0, 5, 10, 20 μM. (**A**) Measurement of OCRs and time (min) curve. (**B**) Analysis of basal respiration. (**C**) Analysis of ATP production. (**D**) Analysis of proton leak. (**E**) Analysis of maximal respiration capacity. (**F**) Analysis of spare respiration capacity. (**G**) Analysis of non-mitochondrial respiration. (**H**) Analysis of extracellular acidification rate. The data are shown for a minimum of three independent trials in triplicate analyzed using Student’s *t*-test to determine significance; ** *p* < 0.01 relative to the stellettin B untreated groups.

A further investigation of stellettin B in regulating mitochondrial oxygen consumption in OC2 cells revealed that this compound generally inhibited all respiratory parameters (oxygen consumption rates, OCRs) in a significant manner. Stellettin B at a concentration as low as 5 μM significantly abrogated parameters of basal respiration, ATP production, proton leak, maximal respiration capacity, and spare respiration capacity (Figure 3A–F). In contrast, no significant changes in non-mitochondrial respiration and extracellular acidification rates at the stellettin B concentrations tested (Figure 3G,H). This was the first time to use the seahorse energy metabolism instrument to observe that stellettin B could affect mitochondrial and aerobic respiration but not glycolysis and anaerobic respiration in OC2 cells.

### 2.4. Stellettin B Induces ER Stress

**Figure 4:**
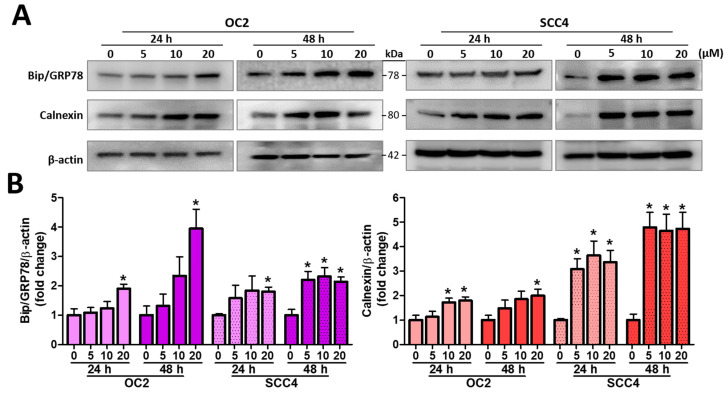
The effects of stellettin B on the levels of ER stress-related protein biomarkers calnexin and BiP/GRP78. OC2 and SCC4 cells were exposed to 0–20 µM stellettin B for 24 and 48 h. (**A**) Western blot analysis of the expression of ER stress proteins BiP/GRP78 and calnexin. (**B**) Quantified levels of BiP/GRP78 and calnexin. Original, uncropped Western blots images are displayed in Supplementary Figure S2. β-actin was used as an internal control. The data are shown for a minimum of three independent trials in triplicate analyzed using Student’s *t*-test to determine significance; * *p* < 0.05 relative to the stellettin B untreated groups.

A subsequent experiment was conducted to investigate the association of stellettin B-induced ER stress, and the expression levels of ER stress-related proteins calnexin and binding immunoglobulin protein (BiP)/GRP78 were measured and monitored (Figure 4A). As displated in Figure 4B, the expression levels of calnexin were all significantly increased in both cells after 24 and 48 h treatment at 20 µM of stellettin B, with fold changes higher than 1.8. Similarly, for the expression levels of BiP/GRP78, they were all significantly increased in both cells after 24 and 48 h treatment at 20 µM of stellettin B, with fold changes higher than 1.7. Taken together, stellettin B stimulated the ER stress in OSCC cells.

### 2.5. Stellettin B Induces MAPK Activation

**Figure 5:**
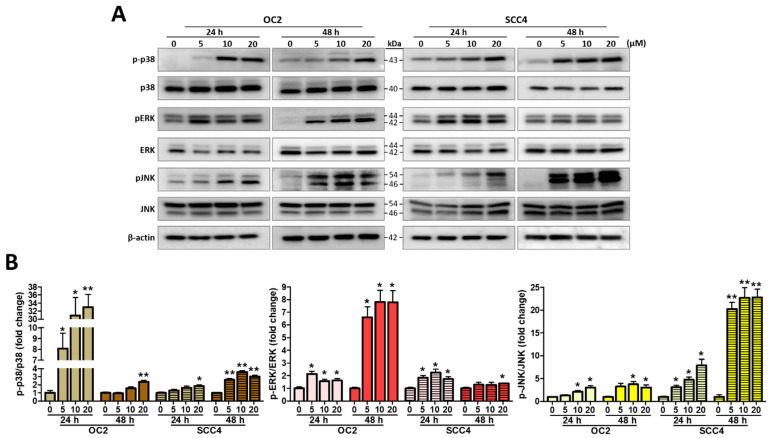
Stellettin B regulated MAPK signaling. (**A**) Western blotting for p38, p-p38, ERK, pERK, JNK, and pJNK levels in OC2 and SCC4 cells treated with 0–20 µM stellettin B for 24 and 48 h. (**B**) Quantification of the MAPKs. Original, uncropped Western blot images are displayed in Supplementary Figure S3. β-actin was blotted as an internal control, and error bars stand for the mean ± SE. The data are shown for a minimum of three independent trials in triplicate analyzed using Student’s *t*-test to determine significance; * *p* < 0.05 and ** *p* < 0.01 relative to the control (untreated cells).

The MAPK signals are activated in response to cell cycle progression and cell survival or death signaling including various cellular stresses including the ER stress [26]. We also analyzed the expression levels of MAPK signaling-related proteins in their phospho-active and non-active forms, which included c-Jun N-terminal kinase (JNK), p38, and extracellular signal-regulated kinase (ERK) and their phosphorylated forms pJNK, p-p38, and pERK. After 24 and 48 h of OC2 and SCC4 cells with stellettin B at various concentrations, p-p38, pERK, and pJNK were increased in their expression levels, demonstrating that the three proteins were activated (Figure 5A). We also found dose dependency for these MAPKs, as evidenced by their increasing expression levels with increasing stellettin B concentration, and they were all significantly increased at 20 µM (Figure 5B). Taken together, both OSCC cell lines were affected by stellettin B that caused alterations in pERK, pJNK, and p-p38 expression in response to the ER stress.

### 2.6. Stellettin B Induces Autophagy-Related Acidic Vesicular Organelles and Autophagic Protein Expression

**Figure 6:**
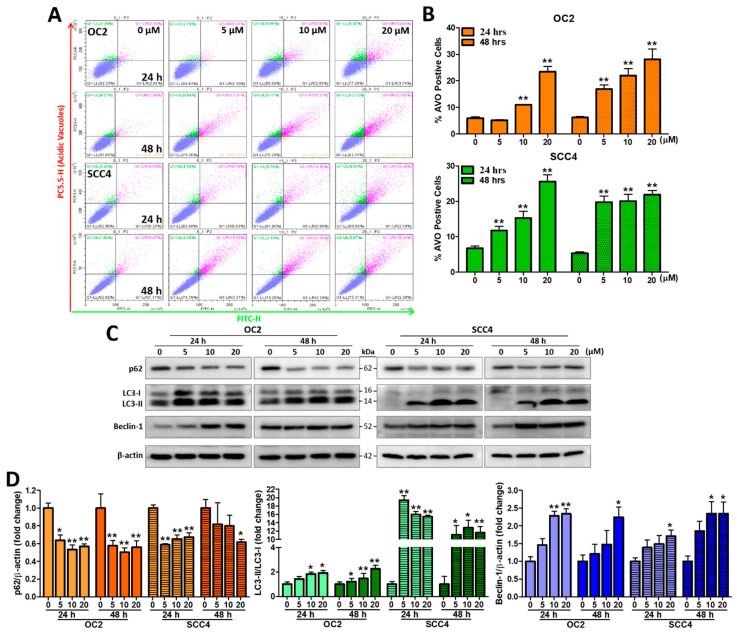
The effects of stellettin B on autophagy-related AVOs and autophagy proteins p62, LC3-I/II, and Beclin-1. (**A**) The fluorescence intensity of AO measured for detecting autophagy at 0–20 µM stellettin B for 24 and 48 h using a Beckman CytoFLEX flow cytometer. (**B**) Quantified levels of AVO-positive cells after exposure to 0–20 µM stellettin B for 24 and 48 h were analyzed using Beckman CytoExpert flow analysis software (v.2.5., Indianapolis, IN, USA). (**C**) OC2 and SCC4 cells were exposed to 0-20 µM stellettin B for 24 and 48 h. Western blot analysis was performed with the use of antibodies against autophagy-related proteins LC3, p62, Beclin-1, and β-actin. (**D**) Quantified levels of the proteins in Figure 3C. Original, uncropped images of the Western blots are displayed in Supplementary Figure S1B. β-actin was used as an internal control, and error bars stand for the mean ± SE. The data are shown for a minimum of three independent trials in triplicate analyzed using Student’s *t*-test to determine significance; * *p* < 0.05 and ** *p* < 0.01 relative to the control (untreated cells).

Previous studies have reported that anticancer drugs may induce cancer cell apoptosis with autophagy concurrently activated [27]. Therefore, we evaluated whether stellettin B could induce autophagy. The formation of acidic vesicular organelles (AVOs) is a characteristic presentation, which is detected using acridine orange (AO). The AO-stained cytoplasm and nucleus emit slightly red and green fluorescence, respectively, while the acidic compartments show bright-to-red fluorescence. A degree of red fluorescence intensity indicates a positive correlation with acidity [28,29]. OC2 and SCC4 cells were treated with 0, 5, 10, and 20 µM stellettin B for 24 and 48 h. As shown in Figure 3, stellettin B treatment at 10 and 20 µM were able to significant induce AVOs in OC2 at 24 and 48 h compared with untreated cells, while stellettin B treatment at 5 µM and above could significant induce AVOs in SSC4 cells (Figure 6A,B). The protein levels of autophagy-related biomarkers LC3 and Beclin-1 were all significantly increased at 20 µM at 24 and 48 h with fold changes higher than 2.0. In contrast, the levels of p62 were all significantly decreased at 20 µM with fold changes lower than 0.7 (Figure 6C,D). Collectively, these results showed that stellettin B induced autophagy-related acidic vesicular organelles and affected proteins related to autophagy cell death in both OSCC cells.

### 2.7. The Autophagy Inhibitor 3-MA Attenuates Stellettin B-Induced Cell Viability and Autophagic Proteins

**Figure 7:**
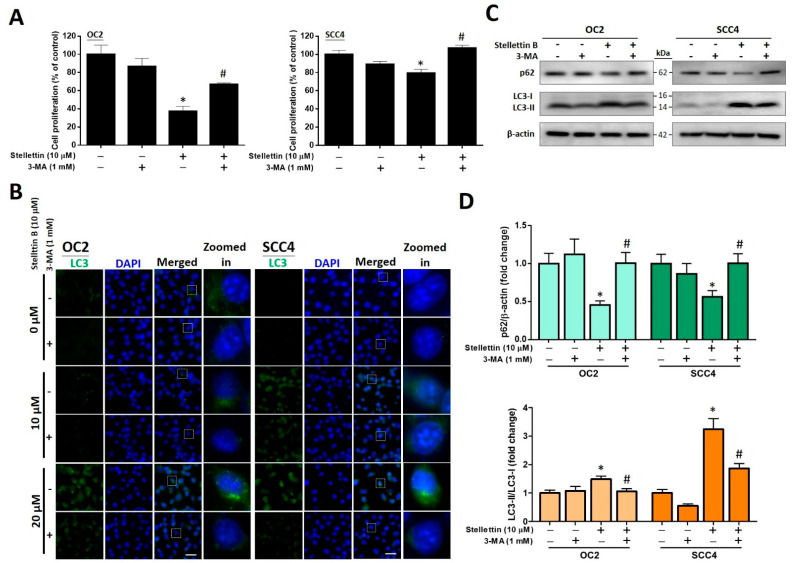
The rescue effects of the autophagy inhibitor, 3-MA, on stellettin B-induced cell viability and autophagic proteins. (**A**) OC2 and SCC4 cells were pre-exposed to 1 mM 3-MA prior to exposure to 10 µM stellettin B for 48 h. Cell viability was determined using MTT-based assay. (**B**) Autophagy was evaluated by conducting immunofluorescence staining of LC3 proteins (showing green signal), while the nuclei were labeled with DAPI (showing blue signal). Scale bar = 50 μm. On the merged images, the white squared areas are enlarged and placed on the right shown as zoomed-in. (**C**) Western blotting for autophagy-related proteins p62 and LC3-I/II in OC2 and SCC4 cells after 48 h treatment. (**D**) Quantified levels of p62 and LC3-I/II. Original, uncropped Western blot images are displayed in Supplementary Figure S4, and error bars stand for the mean ± SE. The data are shown for a minimum of three independent trials in triplicate analyzed using Student’s *t*-test to determine significance; * *p* < 0.05 and # *p* < 0.05 relative to the 3-MA untreated and stellettin B untreated groups, respectively.

The chemical 3-methyladenine (3-MA) acts as an autophagy inhibitor targeting class III phosphatidylinositol 3-kinases (PI3Ks), preventing the formation of autophagosomes. Using MTT assay, we analyzed whether 3-MA could reverse stellettin B-induced cell death after 48 h treatment. The results showed that 1 mM 3-MA significantly enhances cell survival after 10 µM stellettin B treatment in both cell lines (Figure 7A), indicating that autophagy could play a role in stellettin B-induced cell death. We also pretreated OC2 and SCC4 cells with 1 mM 3-MA for 1 h, followed by 48 h treatment with 0, 10, and 20 µM stellettin B, and measured the expression levels of the autophagy-related protein LC3 using immunofluorescence staining. LC3 puncta accumulation induced by stellettin B was ablated by 3-MA (Figure 7B). Further probing with Western blot analysis suggested that the expression levels of p62 were decreased at the fold changes of 0.45 ± 0.09 and 0.6 ± 0.1 in OC2 and SCC4 cells, respectively, while LC3-II/LC3-I ratios were increased at the fold changes of 1.5 ± 0.2 and 3.2 ± 0.6 in OC2 and SCC4 cells, respectively. With the use of 1 mM 3-MA pretreatment, stellettin B-induced downregulation of p62 and upregulation of LC3-II/LC3-I ratios were reversed to their corresponding original levels (Figure 7C,D). Collectively, these findings further suggest that stellettin B could induce autophagy.

## 3. Discussion

**Figure 8:**
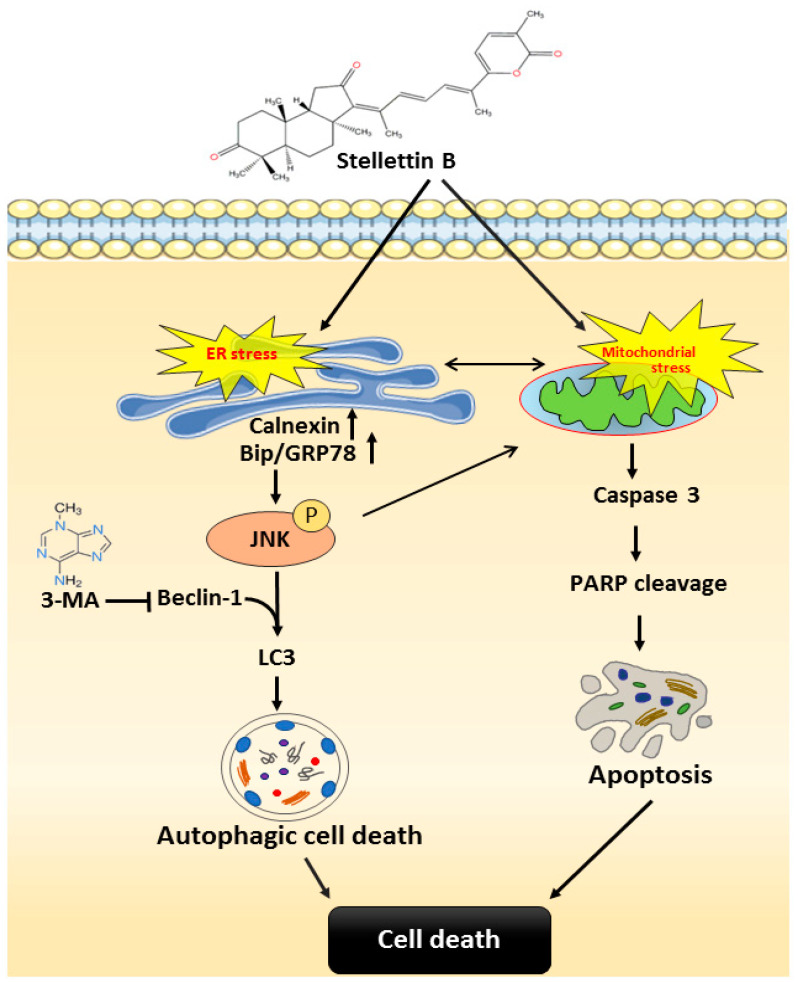
Proposed mechanisms of stellettin B-induced anti-oral cancer effect.

Despite marked improvement in OSCC treatments, delayed diagnosis usually leads to invasion of cancer cells into the oral cavity, metastasis, and/or recurrence. This results in a ~50% 5-year survival rate among OSCC patients. In this study, we demonstrated that the marine-derived natural product stellettin B is active against OSCC. More specifically, stellettin B induces apoptosis and autophagic events in the OSCC cell lines OC2 and SCC4 by regulating ER stress, mitochondrial stress and MAPK signaling. Proposed mechanisms for stellettin B-induced anti-cancer effects are displayed in Figure 8. As shown in Figure 8, stellettin B induces the ER stress which subsequently causes activation of calnexin and Bip/GRP78. Then, Bip/GRP78-mediated JNK phosphorylation up-regulates Beclin-1. LC3 level is also up-regulated, leading to autophagic cell death. However, the autophagy is reversed with 3-MA. Stellettin B induces mitochondrial stress that causes activation of caspase 3. The activated caspase 3 subsequently mediates PARP cleavage to induce apoptosis. Both apoptosis and autophagy lead to cell death as a result.

Induction of mitochondrial dysfunction has recently been demonstrated as an alternative therapeutic strategy for cancer therapy. Given that tumors grow rapidly and have high energy demands compared to surrounding normal tissues, energy metabolism provides a potential therapeutic target for cancer. Interestingly, contrary to the Warburg effect, recent studies have shown that tumor cells in nutrient-compromised environments depend on mitochondrial metabolism for energy metabolism and survival [30]. Inhibition of mitochondrial function could therefore target not only tumor cells but also quiescent tumor stem cells [31].

A number of studies have shown that stellettin B can induce apoptosis and autophagy-associated cytotoxicity in multiple cancer types, such as glioblastoma, lung cancer, and leukemia [32]. In the glioblastoma cell line SF295, stellettin B caused the production of reactive oxygen species (ROS) and increased caspase 3/7 and PARP activities, which ultimately led to apoptosis [20]. In addition, our previous research showed that stellettin B inhibits the invasion, migration, and angiogenesis of glioblastoma cells by attenuating vascular endothelial growth factor secretion [18]. In the non-small-cell lung cancer cell line A549, stellettin B ablated the expression of cyclin D1 and phosphorylated Rb and upregulated p27 levels, leading to G1 phase arrest, an increase in cleaved PARP levels, ROS overproduction, and apoptosis [21]. In the chronic myeloid leukemia K562 cell line, stellettin B increased the expression levels of Bad and Bax and decreased Bcl-2 levels. Additionally, stellettin B activated caspases 9/3 and PARP, caused ROS overproduction, and disrupted mitochondrial function, ultimately promoting apoptosis [23]. Although stellettin B has been studied in some cancer types, its effects on OSCC had not been investigated. In this study, we applied stellettin B to two OSCC cell lines, and our results (Figure 2) are consistent with previous studies showing that stellettin B induces apoptosis by upregulating cleaved caspase 3 and cleaved PARP [20,21,23]. In addition, we found that the activation of caspase 3 and PARP is likely to be induced by mitochondrial dysfunctions (Figure 7A–F), since multiple studies have shown that damaged mitochondrial respiration is associated with abnormal energy metabolism and apoptosis induction [4,5,6]. This conclusion of mitochondria-associated apoptosis is also buttressed by the findings showing no impacts on non-mitochondrial energy metabolism such as glycolysis (Figure 7G,H).

In addition to the apoptosis-associated anticancer mechanism, stellettin B has also been found to be involved in the generation of oxidative stress, cell cycle arrest, and autophagic cell death [32]. A previous study reported that the stellettin B isomer stellettin A could induce abnormal protein glycosylation and upregulate the chaperone protein GRP78, resulting in ER stress, autophagy, and, ultimately, the growth of murine melanoma B16 [33]. ER is a critical intracellular organelle that is responsible for folding and modifying secretory proteins and plays a vital role in regulating calcium homeostasis and lipid metabolism [34]. When a cell is stimulated or under stress, such as dysregulation of calcium homeostasis, environmental toxins, and oxidative stress, ER stress could occur due to the accumulation of unfolded or misfolded proteins in the ER [35]. Studies have shown that ER stress is associated with cancer cell apoptosis, and anticancer compounds can cause apoptosis by inducing ER stress [36]. In this study, we found that stellettin B-induced ER stress and increased the expression levels of calnexin and GRP78. These results indicate that ER stress was enhanced (Figure 4). Interestingly, GRP78 has been found to be a critical regulator of not only ER stress but also autophagy [37]. ER stress and autophagy are two systems dynamically interconnected. Autophagy is a lysosome-involved degradation pathway in a cell responsible for degrading organelles and proteins, and thus, it can be activated by ER stress as an important protective mechanism in cells [10]. Mild- and moderate ER stress is usually a compensatory mechanism in a cell, whereas severe and chronic ER stress hampers cellular functions, resulting in the initiation of cell death signaling [9,35]. Studies have shown that several anti-OSCC chemotherapeutics, such as the proteasome inhibitor bortexomib and the protein kinase B–mammalian target of rapamycin (AKT-mTOR) inhibitor alkylphosphocholine erufosine induce autophagic cell death by increasing ER stress and intracellular ROS [38,39]. In this study, we found that stellettin B induces ER stress in OSCC cells (Figure 4), which ultimately leads to apoptosis and autophagic cell death.

Autophagy in a cell acts as a “double-edged sword” that can both promote tumor growth (protective autophagy) and inhibit cell survival (autophagic cell death) [40]. In a previous study, stellettin B upregulated the expression levels of cleaved PARP and altered those of autophagy-related proteins LC3B II/I, Atg5, and p62, thereby contributing to autophagy and apoptosis [21]. p62 is an autophagic cargo adapter responsible for recruiting ubuquitinated proteins and organelles and forming autophagosomes for degradation. During the autophagic process, Beclin-1 and LC3-II promote the formation of autophagosomes [10]. In this research, we found that stellettin B decreases the expression levels of p62 and increases Beclin-1 and LC3-II levels (Figure 3). Additionally, stellettin B induces OSCC cell death (Figure 1). Studies have reported that polyphyllin G and resveratrol induce cell death via the autophagic pathway in OSCC [41,42], and stellettin B induces autophagic cell death in the lung cancer cell line A549 [21]. These reports therefore support the fact that stellettin B induces autophagic cell death in OSCC cells. In this study, stellettin B-induced autophagic cell death in OSCC cells was validated with the use of 3-MA (Figure 6). Accordingly, we believe that autophagy is the main pathway by which stellettin B causes cell death of OSCC cells.

While the mechanism of action of stellettin B in cancer has been reported earlier, its effects on MAPK signaling have not been not described. MAPK signaling is recognized to be involved in cell proliferation, differentiation, migration, and death [43], and ERK 1/2, p38, and JNK are the main MAPKs [44]. MAPK activation is believed to be involved in the induction of ER stress that causes cell death [26]; in particular, p38 and JNK are known as stress-activated protein kinases induced in response to cellular stress for regulating cell death and survival [15]. In gingival fibroblasts cells, p38 activation is involved in the generation of ER stress that induces autophagy [45]. JNK is a studied regulator of stress-induced apoptosis [46], which is consistent with the finding that it is a tumor suppressor in OSCC [47]. In addition, JNK activation is downstream of autophagy and dependent on the autophagic process [48]. ERK is principally stimulated by growth factors and is found to promote cell proliferation and differentiation [44]; however, cell death induced by the anticancer drugs resveratrol and quercetin cause abnormal changes in ERK activity [49,50]. In this research, we found that stellettin B activates p38, JNK, and ERK in OC2 and SCC4 cells (Figure 5), which is consistent with previous studies. Kim et al. reported that *N*-(4-hydroxyphenyl)retinamide-induced apoptosis is modulated by activation of JNK, p38, and ERK via ROS induction in head and neck squamous carcinoma [51]. JNK, p38, and ERK activation was also observed in polyphyllin G-induced autophagy and apoptosis in OSCC and nasopharyngeal carcinoma [41,52]. Therefore, we cannot rule out the fact that the anticancer effects of stellettin B are mediated by MAPK signaling-involved cell death, although further studies are needed to understand whether stellettin B can induce MAPK (JNK, p38, and ERK)-associated autophagic cell death.

Stellettin B is a marine sponge-derived malabaricane-type triterpene that was first isolated from *J. stellifera* [16,17]. Malabaricane-type triterpenes have been found in various organisms. The first malabaricanes of this family were isolates of the tree *Alianthus malabarica*, whose natural products are yellow pigments, as characterized by a tricyclic terpenoid core and a conjugated polyene side chain [53,54,55]. The identified compound, stellettin B, is categorized as an isomalabaricane-type triterpene. Different from malabaricanes, isomalabaricanes are found in various marine sponges, with a *trans-syn-trans* conformation in the tricyclic ring instead of *trans-anti-trans* [56]. To date, marine sponges have been shown to produce many bioactive natural compounds with pharmacological potential. In 2018, ~28,000 new compounds were isolated from marine organisms, with more than 30% of the new marine-derived natural products obtained from sponges [57,58]. However, sustainable supply has been a critical limitation for the research and development of marine-derived compounds. For example, for future development or industrial use, obtaining these bioactive substances from the wild will impact the marine ecosystem. Aquaculture of potential marine organisms may resolve the above circumstances and may prompt drug discovery and industrial application. At present, marine sponges are transplanted and cultured in cultivating tanks at the National Museum of Marine Biology and Aquarium (NMMBA), Taiwan, by which the sustainability issue could be resolved.

## 4. Materials and Methods

### 4.1. Reagents

The *J. stellifera* sponge was a kind gift sent by Professor Jui-Hsin Su and Professor Ping-Jyun Sung (National Museum of Marine Biology and Aquarium, Taiwan). Stellettin B was an isolated product of the marine sponge and it was dissolved in dimethyl sulfoxide (DMSO) to be a stock. 3-MA was a commercial product from Cayman (Ann Arbor, MI, USA). MTT, 4,6-diamidino-2-phenylindole (DAPI), and the hydrophilic detergent Tween 20 were commercial products from Sigma-Aldrich (St. Louis, MO, USA). Protease and phosphatase inhibitor cocktail tablets were commercial products from Roche Diagnostics (Mannheim, Germany). The DC protein assay kit was a commercial product from Bio-Rad Laboratories (Hercules, CA, USA). The Immobilon™ Western chemiluminescent horseradish peroxidase (HRP) substrate was a commercial product from Millipore (Billerica, MA, USA).

### 4.2. Cell Culture

The human OSCC cell line OC2, derived from squamous carcinoma of the buccal mucosa, was obtained from Veterans General Hospital (Taipei, Taiwan). OC2 cells were cultured in Roswell Park Memorial Institute (RPMI) 1640 medium (Gibco BRL, Rockville, MD, USA). The human OSCC cell line SCC4, sampled from squamous cell carcinoma in the tongue, was a commercial product from the Bioresource Collection and Research Center based in Hsinchu, Taiwan, and cultured in Dulbecco’s Modified Eagle Medium (DMEM)/F12 medium (Gibco) with 400 ng/mL of hydrocortisone. Both media contained 10% heat-processed fetal bovine serum (Millipore) and 100 units/mL of antibiotic penicillin and 100 µg/mL of antibiotic streptomycin (Gibco). A humidified atmosphere supplemented with 5% CO_2_ was generated for culturing the above cells.

### 4.3. Crystal Violet Assay

The cell viability of OC2 and SCC4 was evaluated with the use of the crystal violet assay. Briefly, 5 × 10^3^ cells per well were seeded in transparent 96-well plates and exposed to various concentrations of stellettin B for 24 and 48 h. After stellettin B exposure, culture medium was removed from plate wells which were then washed with PBS. A solution containing 0.5% crystal violet and 1% formalin was added to each well, and incubated for a total of 10 min at room temperature. The crystal violet solution was then aspirated and washed with tap water. A mixture solution of 0.01% acetic acid in 50% ethanol was added to solubilize the crystal violet stain. Finally, absorbance was fixed at 570 nm for recording using a microplate spectrophotometer (BMG LABTECH, Offenburg, Germany).

### 4.4. Cell Viability Assay: MTT Method

The viability of OC2 and SCC4 cells was evaluated by the MTT-based assay. Briefly, 5 × 10^3^ cells per well were seeded in transparent 96-well plates and exposed to various concentrations of stellettin B for 24 and 48 h. After stellettin B exposure, the treated cells were stained by MTT solution. Finally, absorbance was fixed at 570 nm for recording using a microplate spectrophotometer (BMG LABTECH, Offenburg, Germany).

### 4.5. Cell Cycle Analysis

Cell cycle analysis for the OSCC cells was completed, as described in the literature [59]. OC2 cells were exposed to 0–40 µM stellettin B for 24 h, harvested, washed with PBS, and centrifuged for subsequent fixation with 100% ethanol at 4 °C for 2–4 h. The fixed cells were then stained with a PBS solution containing 50 μg/mL of PI and RNase A. Afterwards, the cell cycle events were analyzed using a Beckman Coulter flow cytometer (Beckman Coulter, MI, USA) equipped with Cell Lab Quanta™ SC analysis software v.1.0. A number of 10,000 cells per sample, or higher, was analyzed.

### 4.6. TUNEL Staining

TUNEL staining was performed, as described in the literature [4,5]. After exposure to stellettin B, apoptotic OC2 and SSC4 cells were studied using a commercial in situ cell death detection kit (Roche Life Science, Penzberg, Upper Bavaria, Germany). The OSCC cells were seeded on sample coverslips, exposed to stellettin B for 48 h, and fixed in 4% paraformaldehyde in PBS for 1 h at room temperature. Next, the cells were incubated with a permeabilization solution containing 0.1% Triton X-100 and 0.1% sodium citrate at 4 °C for 10 min. Next, a TUNEL reaction buffer was added at 37 °C for 1 h, while DAPI was incubated for 10 min at room temperature; both dyes were used in the dark. A Leica TCS SP5 DM 6000 CS fluorescence microscope (Leica, Wetzlar, Germany) was used for observation, and the SPOT Xplorer image capture system (Diagnostic Instruments, Sterling Heights, MI, USA) was used to capture images.

### 4.7. Western Blot Analysis

OC2 and SSC4 cells were exposed to stellettin B for 24 or 48 h, and then harvested and lysed with RIPA buffer supplemented with a commercial protease and phosphatase inhibitor cocktail (Roche Diagnostics). Then, cell lysates were centrifuged at 13,000 rpm for 30 min at 4 °C for collecting the supernatant for Western blot analysis. Protein concentrations were estimated using a commercial DC protein assay kit (Bio-Rad Laboratories). Blotting wells were loaded with equal amount of total proteins for electrophoresis, followed by transferring to polyvinylidene difluoride (PVDF) membranes (Millipore). The PVDF membranes were blocked with 5% skim milk that was dissolved in Tris-buffered saline, 0.1% Tween 20 detergent (TBST) for 1 h at room temperature, incubated overnight with primary antibodies at 4 °C, and then incubated with HRP-conjugated secondary antibodies for 1 h at room temperature. Proteins of interest were detected by enhanced chemiluminescence (Millipore). Image acquisition and following quantification were completed using the BioSpectrum 610 Imaging System and VisionWorks^®^ LS software (Wasserburg, Germany). The primary antibodies used in this research were p-ERK, ERK, p-P38, P38, p-JNK, JNK, BiP/GRP78, calnexin, p62, LC3, and Beclin-1, as shown in Table 1. HRP-conjugated anti-rabbit immunoglobulin G (IgG) secondary antibody was a commercial product from Jackson ImmunoResearch Laboratories, Inc. (West Grove, PA, USA).

### 4.8. Immunofluorescence Staining

OC2 and SSC4 cells were seeded onto sample coverslips and exposed to stellettin B for 48 h. After exposure, the cells were fixed in cold methanol on ice for 10 min, and then permeabilization solution (0.1% Triton X-100 in PBS) was added at 4 °C for 10 min, followed by adding blocking solution, that is, 1% bovine serum albumin together with 0.1% Tween 20 in PBS, and shaking for 1 h at room temperature. The primary antibody, LC3, was diluted at 1:100 in the antibody diluent (1% BSA together with 0.1% Tween 20 in PBS) and incubated at 4 °C for 16–18 h. The secondary antibody, Alexa Fluor 488 anti-rabbit IgG, was diluted at a ratio of 1:200 and incubated at room temperature and protected from light for 2 h. Nuclei were visualized with DAPI stain for 10 min at room temperature in the dark. A Leica TCS SP5 DM 6000 CS fluorescence microscope (Leica) was used for observation, and the SPOT Xplorer image capture system (Diagnostic Instruments) was used to capture images.

### 4.9. Quantification of AVOs

The following procedure was referenced from the literature [6]. OC2 and SSC4 cells were prepared at 2 × 10^5^ cells per well in transparent 6-well plates (ThermoFisher, Rochester, NY, USA). Following overnight incubation, the cells were exposed to specified concentrations of stellettin B for 24 and 48 h. Next, the cells were washed twice with PBS, after which 1 µg/mL of AO reagent was added at 37 °C for 20 min. The cells were harvested and PBS-washed and finally diluted into 0.5 mL PBS. The Beckman CytoFLEX flow cytometer was used for evaluating the data from the AVO assay, using Beckman CytoExpert flow analysis software. A number of 10,000 cells per sample, or higher, was analyzed.

### 4.10. Mitochondrial Respiration Measurement

The following procedure was referenced from the literature [6]. A Seahorse XF24 Extracellular Flux Analyzer provided by Seahorse Bioscience (Chicopee, MA, USA) was used for mitochondrial consumption of oxygen in a cell. The OC2 cells were seeded in 24-well plates (1 × 10^5^ cells/well) and placed in the 37 °C incubator for 18 h. The culture media were then replaced with stellettin B-supplemented medium for 24 h of incubation. Following rinsing the cells with 1 mL of sodium bicarbonate-free DMEM, 675 μL of the DMEM were added to each well. Four values of the basal OCR were averaged under basal conditions. Subsequently, 1 μM of oligomycin, 250 nM of FCCP, and 2 μM of rotenone were sequentially added. A standard curve of protein concentration was generated using bovine serum albumin with a DC protein assay kit (Bio-Rad, CA, USA). Data were expressed as OCR in pmol/min/mg protein and ECAR in mpH/min/mg protein, and were calculated after normalization with protein concentration.

### 4.11. Statistical Analysis

The SPSS software (Windows 13.0 version; SPSS Inc., Chicago, IL, USA) was used for all statistical analyses. Data displayed in this study are shown as the mean ± standard error (SE), and they were analyzed using Student’s *t*-test with *p* < 0.05 considered as a level of statistical significance.

## 5. Conclusions

Overall, this study demonstrated that stellettin B induces apoptosis and autophagy by affecting the ER stress and mitochondrial stress and activating MAPK signaling, which shows potential to be further developed for use in cancer.

## Figures and Tables

**Table 1:** Information about primary antibodies used in Western blot analysis in this study.

Primary Antibody	Host	Catalog #	Supplier	Dilution Ratio
Cleaved caspase-3	Rabbit	9664S	Cell Signaling	1:1000
PARP	Rabbit	9542S	Cell Signaling	1:1000
p62/SQSTM1	Rabbit	18420-1-ap	ProteinTech	1:1000
Beclin-1	Rabbit	11306-1-ap	ProteinTech	1:1000
LC3	Rabbit	AP1802a	Abgent	1:1000
Bip/GRP78	Rabbit	ab21685	Abcam	1:1000
Calnexin	Rabbit	ab22595	Abcam	1:1000
Phospho-p38	Rabbit	4511S	Cell Signaling	1:1000
p38	Rabbit	9212	Cell Signaling	1:1000
Phospho-ERK	Rabbit	4370S	Cell Signaling	1:1000
ERK	Rabbit	4695	Cell Signaling	1:1000
Phospho-JNK	Rabbit	4668S	Cell Signaling	1:1000
JNK	Rabbit	9252S	Cell Signaling	1:1000
β-actin (HRP conjugate)	Mouse	12262S	Cell Signaling	1:2000

## Data Availability

The data that supports the findings of this study are available in the Supplementary Materials of this article.

## Supplementary Materials

The supporting information can be downloaded at: https://www.mdpi.com/article/10.3390/ijms23158813/s1.

## Abbreviation & Definition

3-MA	3-Methyladenine
AKT	Protein kinase B
AO	Acridine Orange
Atg5	Autophagy related 5
AVOs	Acidic Vesicular Organelles
BiP/GRP78	Binding immunoglobulin Protein/Glucose Regulatory Protein 78
BSA	Bovine Serum Albumin
DAPI	4,6-Diamidino-2-Phenylindole
DMEM	Dulbecco’s Modified Eagle Medium
DMSO	Dimethyl Sulfoxide
ECAR	Extra Cellular Acidification Rate
ER	Endoplasmic Reticulum
ERK	Extracellular signal-regulated kinases
FCCP	Carbonyl cyanide 4-(trifluoromethoxy)phenylhydrazone
HO-1	Heme-oxygenase 1
HRP	Horseradish peroxidase
IC_50_	50% Inhibitory concentration
IgG	Immunoglobulin G
JNK	c-Jun N-terminal kinase
LC3	Microtubule-associated proteins 1A/1B light chain 3B
MAPK	Mitogen-activated protein kinase
mTOR	Mammalian target of rapamycin
MTT	3-(4,5- dimethylthiazol-2-yl)-2,5-diphenyltetrazolium bromide
NMMBA	National Museum of Marine Biology and Aquarium
Nrf2	Nuclear factor erythroid 2-related factor 2
OCRs	Oxygen Consumption Rates
OSCC	Oral Squamous Cell Carcinoma
OXPHOS	Oxidative phosphorylation
PARP	Poly (ADP-ribose) polymerase
PBS	Phosphate buffered saline
PI3Ks	Phosphoinositide 3-kinases
PVDF	Polyvinylidene difluoride
RPMI	Roswell Park Memorial Institute
RIPA	Radioimmunoprecipitation assay
p62/SQSTM1	Ubiquitin-binding protein p62/Sequestosome-1
ROS	Reactive Oxygen Species
SE	Standard error
TBST	Tris-buffered saline, 0.1% Tween 20 detergent
TUNEL	Terminal deoxynucleotidyl transferase dUTP nick-end labeling
