# Supplementary material for: Stellettin B-Induced Oral Cancer Cell Death via Endoplasmic Reticulum Stress–Mitochondrial Apoptotic and Autophagic Signaling Pathway
Source: Int J Mol Sci. 2022 Aug 8;23(15):8813. doi: 10.3390/ijms23158813 (PMC9368952; doi:10.3390/ijms23158813)
Supplement: Supplementary file 1 [file ijms-23-08813-s001.zip › ijms-1837403-supplementary.pdf]

1. Supplementary Figures

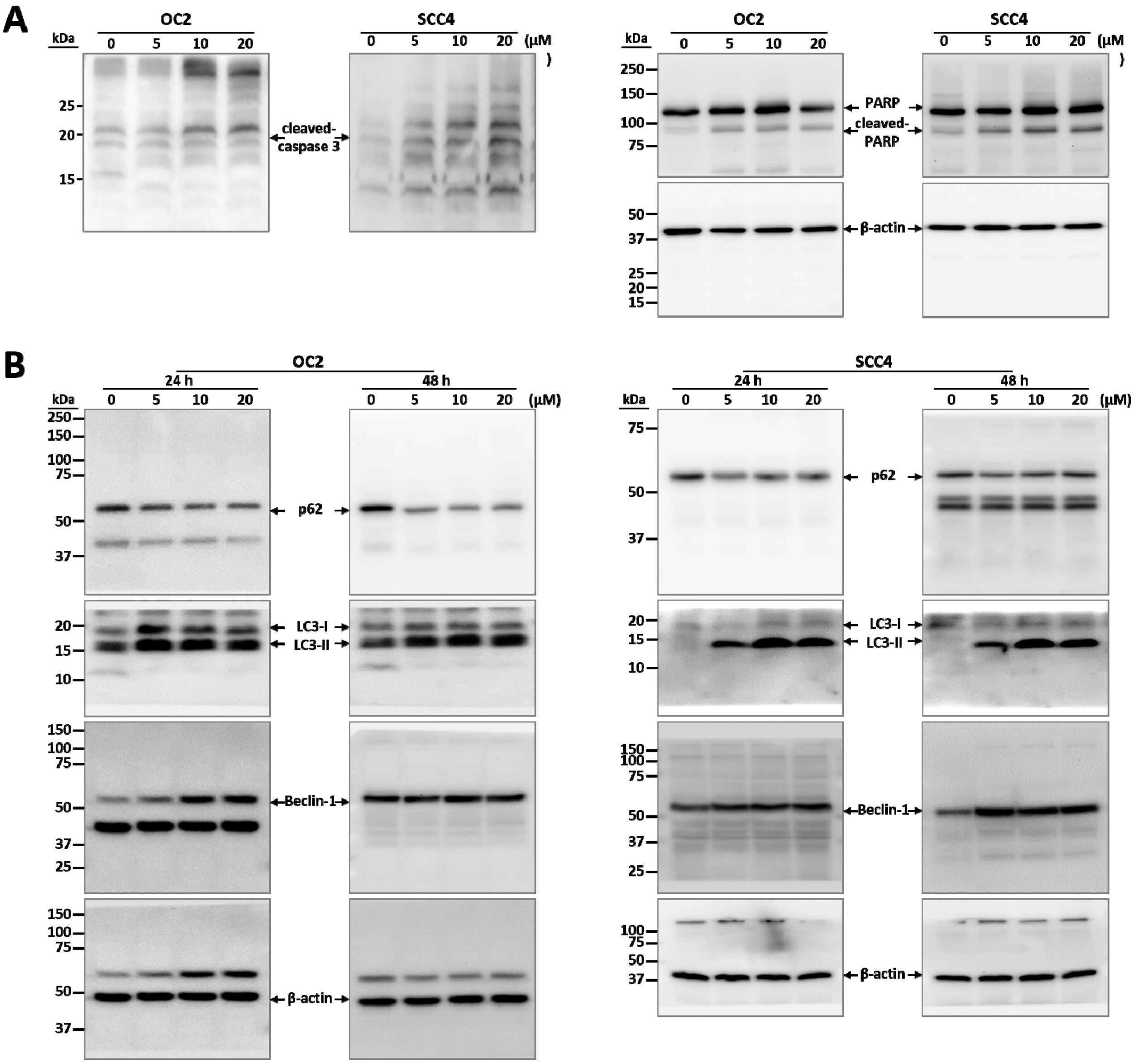

Supplementary Figure S1. Original, uncropped images of the western blots for Fig. 2E and 6C displayed in the text and results. (A) The bands of cleaved caspase 3, PARP and cleaved PARP, and molecular weights of markers, with β-actin used as the protein loading control in OC2 and SCC4 cells. (B) The bands of p62, LC3-I/II, and Beclin-1, and molecular weights of markers, with β-actin used as the protein loading control in OC2 and SCC4 cells.

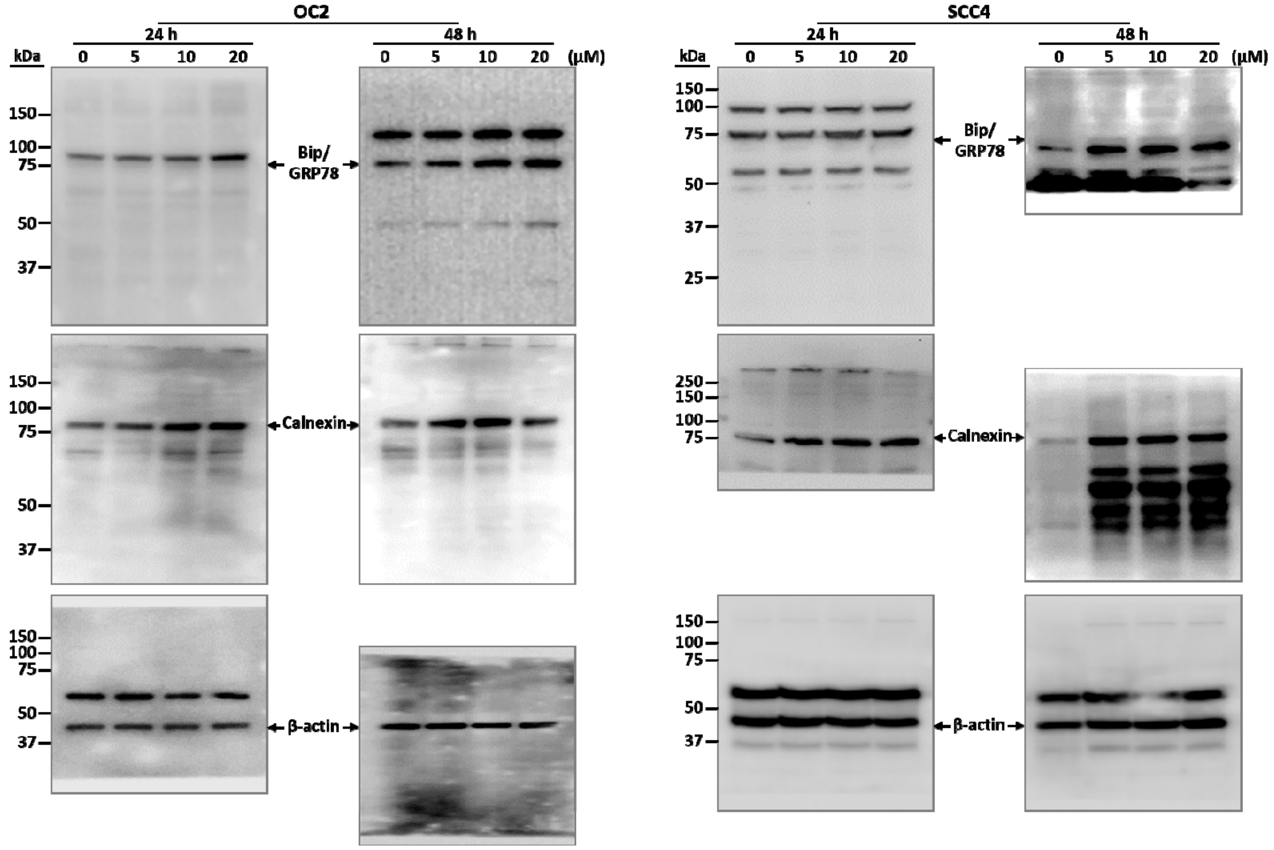

Supplementary Figure S2. Original, uncropped images of the western blots for Fig. 4A displayed in the text and results. The bands of Bip/GRP78 and Calnexin, and molecular weights of markers, with β-actin used as the protein loading control in OC2 and SCC4 cells.

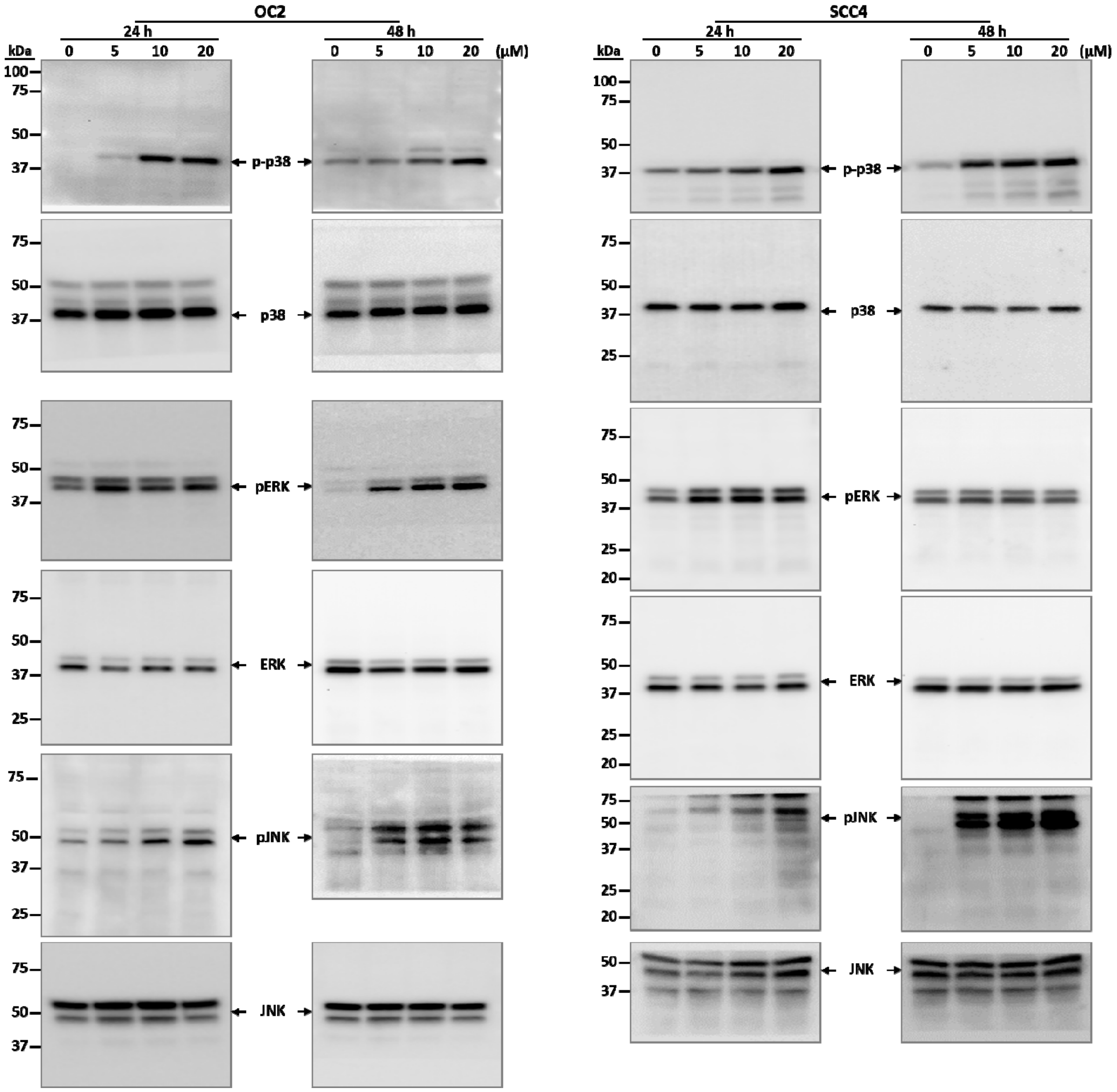

Supplementary Figure S3. Original, uncropped images of the western blots for Fig. 5A displayed in the text and results. The bands of p-p38, p38, pERK, ERK, pJNK, and JNK and molecular weights of markers in OC2 and SCC4 cells.

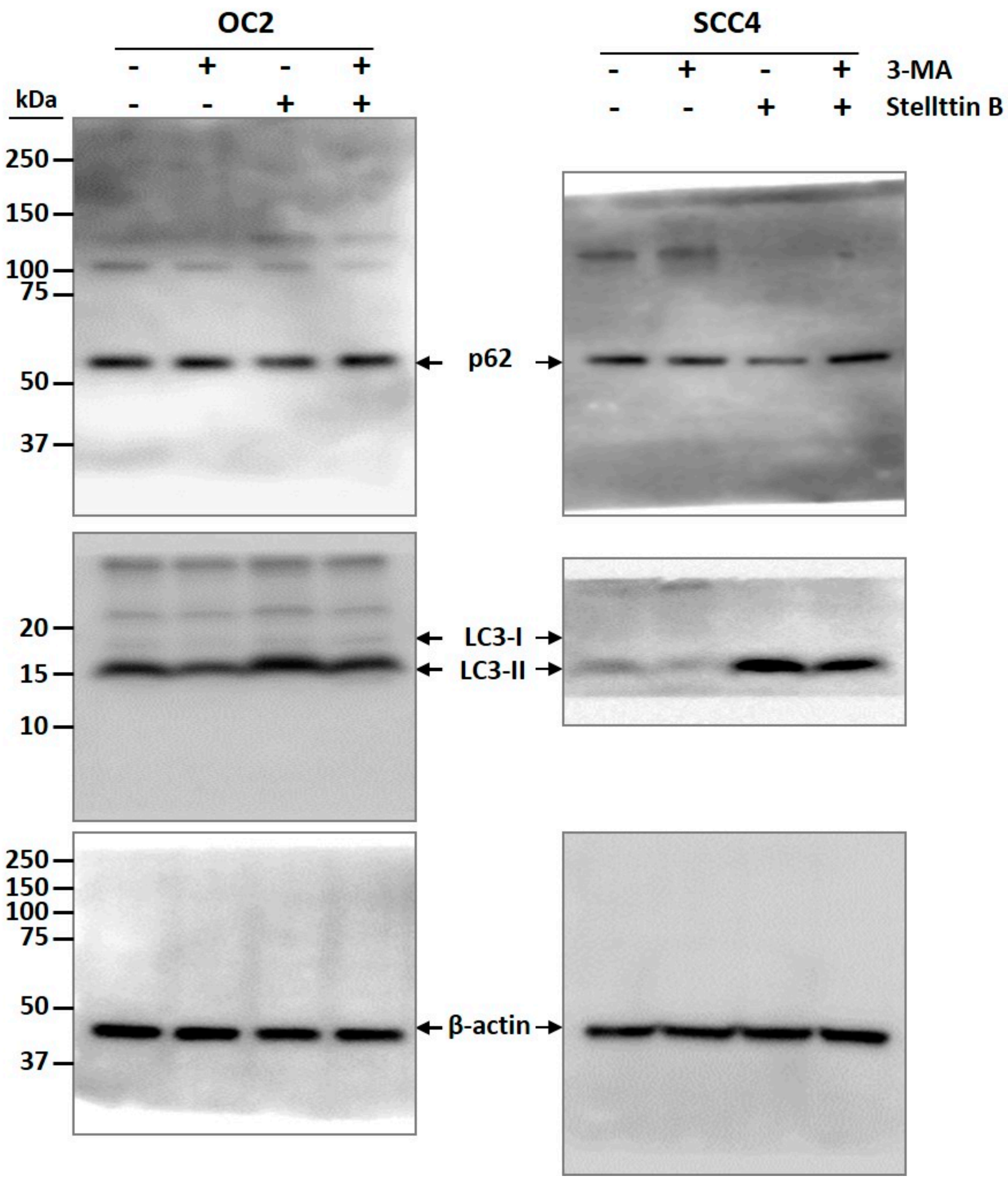

Supplementary Figure S4. Original, uncropped images of the western blots for Fig. 7C displayed in the text and results. The bands of pJNK, JNK, p62, and LC3-I/II, and molecular weights of markers, with β-actin used as the protein loading control in OC2 and SCC4 cells.
